# Prevalence and factors associated with major depressive disorder in children and adolescents at the Uganda Cancer Institute

**DOI:** 10.1186/s12885-019-5635-z

**Published:** 2019-05-17

**Authors:** Benedict Akimana, Catherine Abbo, Joyce Balagadde-Kambugu, Etheldreda Nakimuli-Mpungu

**Affiliations:** 1 Department of Psychiatry, College of Health Sciences, Makerere University, P.O.BOX 7072 Kampala, Uganda; 2 Uganda Cancer Institute, Kampala, Uganda

**Keywords:** Depression, Children, Adolescents, Cancer, Uganda, Out-patient, Prevalence, Low-resource

## Abstract

**Background:**

The prevalence and factors associated with major depressive disorder (MDD) among adults with cancer have been documented in the scientific literature. However, this data is limited among children and adolescents with cancer; especially in low resource settings such as Uganda. We assessed the prevalence and factors associated with MDD in children and adolescents attending the Uganda cancer institute out-patient clinic.

**Methods:**

This was a cross-sectional study in which three hundred and fifty-two children and adolescents with any cancer diagnosis were screened for depression using the Child Depression Inventory (CDI) and evaluated with the mini neuropsychiatric interview for children and adolescents (MINI-KID). Associated factors were assessed using a standardized questionnaire that assessed child and caregiver demographic and psychosocial characteristics. Multiple logistic regression models were used to assess factors independently associated with MDD.

**R**esults**:**

Of the 352 children and adolescents recruited in the study 134(38%) scored above a cut-off point of 13 on the CDI indicating significant depression symptoms. However, 91(26%) met criteria for MDD. The majority of those with MDD (*n* = 59 64.8%) had CDI scores of 13–19 indicating mild depression, 30(33%) had scores of 20–25 indicating moderate depression and 2(2.2%) had scores of 25 and above indicating severe depression. Protective factors against MDD were having a special person in the respondent’s life (*p* = 0.002) and using self-distraction as a coping method (*p* = < 0.001). Risk factors were being an adolescent(*p* = < 0.001).

**Conclusion:**

The prevalence of MDD is substantial in children and adolescents with cancer in Uganda. Given that the majority had a mild-moderate depression, there is an urgent need to integrate psychotherapy-the first-line treatment for depression into the routine care of children and adolescents with cancer.

## Background

Cancer is a major public health problem worldwide, accounting for 8.2 million deaths in 2012. More than 60% of the world’s total new annual cases occur in Africa, Asia and Central and South America. It is projected that cancer cases will rise annually from fourteen million in 2012 to twenty-two million within the next two decades. It is estimated that two hundred thousand children and adolescents suffer from different types of cancer worldwide yearly, with the majority living in low and middle-income countries which translates to a high mortality rate in these countries. [1].

Like any other chronic disease, individuals living with cancer are faced with numerous psychological and social challenges. These challenges are even more pronounced in children and adolescent who have to cope with a number of issues as they move from one stage of development to another [2]. One such challenge is co-morbid mental disorders; with depression being the most common mental health problem [3]. A quarter of patients with cancer suffer from depression, whose symptoms should be looked for and treated by the health workers as they negatively impact the patient’s quality of life [4].

Depression is more prevalent in individuals with physical illnesses than in those without these illnesses; e.g., 33% with cancer, 22% with myocardial infarction, 27% with diabetes, and 29% with hypertension [5]. Cancer patient with depression are usually missed out, this is because the signs and symptoms of depression like weight loss, sleep disturbance, sad mood are mistakenly taken to be due to the medical illness [6]. Thus, serious medical and psychiatric comorbidity is often overlooked, leading to undertreatment that complicates cancer and at the least adversely affects patients’ quality of life [6].

Studies of general psychiatric burden among children and adolescents with cancer estimate the prevalence rates of depression symptoms between 5 and 50% and prevalence rates of depressive disorders between 0 and 46% [7]. Factors that contribute to depression include; patient-related factors such as family history of depression, disease- and treatment-related variables such as pain and other physical symptoms, awareness of cancer diagnosis, and contributions of the patient’s surroundings such as social support [7].

In Uganda, we do not know the extent to which these factors may be associated with depression in children and adolescents with cancer. Most of these studies about the prevalence and factors associated with depression have been done in the adult population, and a limited number of these studies have been specific to children and adolescents.

In the present report, we examine the prevalence of depression, child and caregiver demographics, psychosocial and clinical features associated with having major depression among children and adolescents with any cancer diagnosis. Potential associated factors were selected based on evidence from the pre-existing literature that they are important correlates of depression in populations with chronic disease.

## Methods

### Study setting and population

Study participants were children and adolescents aged 7–17 years recruited from Uganda Cancer Institute (UCI) which is situated at the Mulago National Referral Hospital in Kampala District. It is the sole cancer treatment and training facility in Uganda. Approximately 1200 patients are seen per year 30% being children and adolescents. The child and adolescent clinic operates on Monday, Wednesday and Thursday seeing averagely 20 patients on clinic days. The clinics are run by a team of medical personnel which include the Paediatric oncologist, pharmacist, nurses and support staff.

### Study procedure

Study data were collected between July and December 2015. The eligibility criteria required participants to be present on the days of the interview, an aged 7–17 year with a histological confirmation of active cancer disease and on treatment, provide assent and have parental /guardian written informed consent. On a given clinic day, research assistants (at the level of a nursing assistant who underwent a week-long training in the use of the research tools) under the supervision of the principal investigator worked with clinic staff to obtain a register of clients who had come to the clinic on that day. The clients would be seated in the waiting area awaiting their turn to see the clinician. Research assistants then explained study procedures, determined eligibility and then obtained assent from the client and informed consent from the parent/guardian. The questionnaires were then administered and it took approximately 45 min to complete. The research protocol was approved by the Makerere University School of Medicine Research Ethics Committee, as well as the Uganda National Council of Science and Technology.

### Study measures

#### Socio-demographic variables

Descriptive information including age, gender, orphan status, educational, and income status was assessed using a standardized demographic questionnaire for both children and adolescents as well as the caregivers.

#### Psychosocial variables

Depression was assessed using the CDI [8] followed by the MINI-Kid diagnostic interview this is the children and adolescent version of the Mini-International Neuropsychiatric Interview. It is a short structured diagnostic interview that was developed for DSM-IV psychiatric disorders. The specific modules of the tool that were used in this study were to assess for; MDD [9].

The psychometric properties of the CDI have been assessed in this study. At a cut-off point of 18, the measure attained a sensitivity of 87% and a specificity of 86%. Cronbach alpha was 0.8277 in this study sample diagnostic interview.

Coping skills among the study sample were assessed using the modified coping inventory [10]. A binary variable was created, with the variable coded 1 for endorsing the use of a coping skill and coded 0 for not endorsing the use of the given coping skill. In the analyses coping skills were assessed as a binary variable.

Perceived social support was assessed using four items obtained from the 12-item multi-dimensional social support scale [11], which provides an assessment of three sources of support: family (FA), friends (FR), and significant other (SO). The scale has been validated in Uganda and the three-subscale structure (Family, Friends, and Significant Other) of The Multidimensional Scale of Perceived Social Support was confirmed [12]. The Cronbach α for the four items in this sample was 0.81. Responses were based on a 7-point scale where 1–4 referred to those who very strongly, strongly, mildly disagreed and those who were neutral about having enough support from friends, family and significant others respectively. Scores of 5–7 referred to those who mildly, strongly or very strongly agreed to have enough support respectively. Therefore, a binary variable was created, with the variable coded 1 for an agreement to adequate social support and coded 0 for disagreement or being neutral. Perceived social support was also assessed as a binary variable.

#### Clinical variables

These were assessed from the patient’s medical charts, they included the cancer type, cancer stage, type of treatment, treatment duration (0–1.1–3,> 3 years), HIV status, and family history of depression assessed by a single question and recorded in a clinical data form for each individual.

### Statistical analyses

Statistical analyses were carried out with STATA, version 12. The goal of the analyses was to estimate and identify, among children and adolescents with any cancer diagnosis, the prevalence and factors associated with major depressive disorder. Initially, a binary variable was created for depression, with the variable coded 1 for those who met the MINI criteria for major depressive disorder and coded 0 for those who did not. We used simple logistic regression models to evaluate child and caregiver socio-demographic, psychosocial and clinical variables that were associated with having a major depressive disorder.

Factors that were associated with major depression at a significance level of (*p* ≤ 0.2) were then included in a multivariate logistic regression model. Stepwise logistic regression was used to determine factors independently associated with major depression. We assessed for multicollinearity by computing the variance inflation factor for the variables in the model.

The minimum sample size required for multivariate analysis was estimated using a-priori sample size estimation formula for multiple regression analysis. Given 8 predictors (my associated factors) with significance level (α) set at 0.05, power of 0.8 and anticipated effect size (*f2*) of 0.15, using A-priori Sample Size Calculator 45, a minimum sample size of 108 was required in order to detect a significant model (F (9,104) =1.97). The study sample size of 352 allowed for multiple logistic regression analysis (13). A *p*-value of ≤0.05 was considered statistically significant.

## Results

### Child and adolescent characteristics

#### Socio-demographic variable

Of the 352 children and adolescents, 240 (68.18%) were male and 112 (31.82%) were females, giving a male: female ratio of 2.1:1. Their ages ranged from 7 to 17 years with the mean age being 11.5 years (Standard deviation = 3.2). Other socio-demographic characteristics are presented in Table 1.

**Table 1:** Socio-demographic, clinical and psycho-social variables of children and adolescents with Cancer at UCI outpatient clinic

Socio-demographic variables	*N* = 352	Percentage%
Age category (years)		
Children (7–9)	115	32.67
Adolescents (10–17)	237	67.33
Gender		
Male	240	68.18
Female	112	31.82
Orphanhood status		
Both parents alive	279	79.30
Single Orphan	61	17.30
Double Orphan	12	3.40
Ethnic group according to region		
Western Uganda	80	22.70
Northern Uganda	66	18.60
Eastern Uganda	72	20.50
Central region	110	31.30
Foreigners	24	6.81
Psychosocial variables		
Negative coping strategies		
Denial		
Yes	141	40.1
No	211	59.9
Self-blame		
Yes	56	15.9
No	296	84.1
Substance use		
Yes	17	4.8
No	335	95.2
Behavioural disengagement		
Yes	173	49.2
No	179	50.8
Positive coping strategies		
Self-distraction		
Yes	250	71
No	102	29
Active Coping		
Yes	329	93.5
No	23	6.5
Use of emotional support		
Yes	301	85.5
No	51	14.5
Use of instrumental support		
Yes	295	83.8
No	57	16.2
Acceptance		
Yes	190	54
No	162	46
Positive reframing		
Yes	223	63.4
No	129	36.6
Use of religion		
Yes	264	75
No	88	25
Perception of social support		
A special person in patient’s life		
Yes	292	83.8
No	60	16.2
Emotional Support from family		
Yes	293	83.2
No	59	16.8
Friends in the patient’s life		
Yes	290	82.4
No	62	17.6
Family help		
Yes	292	83
No	60	17
Clinical variables		
A family history of depression		
Yes	127	36.1
No	225	63.9
HIV/AIDS		
Yes	47	13.4
No	233	86.6
Cancer stage		
I	31	8.8
II	45	12.8
III	80	22.7
IV	49	13.9
Other	150	42.6
Cancer category		
Lymphomas	152	43.2
Leukaemia’s	94	26.7
Embryonal tumours	49	13.9
Primary bone tumours	14	4.0
Others	43	12.2
Treatment duration		
0–12 months	239	68.0
12–36 months	103	29.3
> 36 months	10	2.8

#### Clinical variables

Clinically, the majority of study participants (32%) were diagnosed with Lymphomas. Over one third (36%) of them reported a family history of depression. Data obtained from medical charts indicated that the majority (86.6%) were HIV negative. Other clinical characteristics are presented in Table 1.

#### Psychosocial variables

Both negative and positive coping strategies were employed by the participants, with the majority endorsing the positive coping mechanisms. Details of the coping mechanisms are presented in Table 1.

### Caregiver characteristics

Majority of the caregivers enrolled in the study were female, their ages ranged from 17 to 70 years with a mean age of 38.9 years (SD 8.68). In their household, they had an average of 6 people (SD 8.68) under their care with age range of 2–15. The majority was also low-income earners and had primary education. Detailed baseline characteristics of the caregivers are presented in Table 2.

**Table 2:** Socio-demographic characteristics of caregivers of children and adolescents with cancer at the UCI outpatient clinic

Socio-demographic variables	*N* = 352	Percentage%
Gender		
Male	109	31.0
Female	243	69.0
Marital status		
Single	30	8.5
Married/co-habiting	255	72.4
Divorce/separated	33	9.4
Widowed	34	9.7
Employment		
Yes	287	81.5
No	65	18.5
Relationship with patient		
Parent	203	57.7
Relative (other than sibling/parent)	42	11.9
Sibling	26	7.4
Self (no relative/caregiver)	77	21.9
Other (friend/caregiver)	4	1.1
Number of caregiver’s children		
0	26	7.4
1–3	96	27.3
4–6	132	37.5
> 6	98	27.8
Monthly income		
< 75,000shs	109	7.4
75,000-300,000	173	27.3
300,000-500,000	62	37.5
> 500,000	8	27.8

### Prevalence and factors associated with depression symptoms

Of the 352 participants assessed with the MINI-KID 91 (26%) had a major depressive disorder with the majority having mild depression as shown by the scores of 13–19 on the CDI. Table 3 illustrates the bivariate logistic regression analyses results of the child and adolescent characteristics, and Table 4 illustrates the bivariate analyses results of the caregiver characteristics. Table 5 illustrates the multivariate logistic regression analyses.

**Table 3:** Bivariate logistic regression analyses of children and adolescent characteristics

Characteristic	Depression (*N* = 91) *n* (%)	No depression (*N* = 261) *n* (%)	OR (95% CI)	*P* value
Age				
(7–9 years)	14(15.4)	101(38.7)	1	
(10-17 years)	77(84.6)	160(61.3)	3.47(1.83–6.56)	< 0.001
Region of origin				
Central Uganda	25(27.5)	89(34.1)	1	
Eastern Uganda	25(27.5)	46(17.6)	1.5(0.49–4.77)	0.45
Western Uganda	16(17.6)	67(25.7)	0.85(0.41–1.7)	0.65
Northern Uganda	210(21.9)	47(18)	1.51(0.75–3.0)	0.23
Foreigners	5(5.5)	12(4.6)	1.45(0.47–4.63)	0.49
Orphanhood status				
Not orphan	63(69.2)	180(69.0)	1	
Single orphan	23(37.7)	96(36.8)	2.07(1.11–3.76)	0.01
Double orphan	5(5.4)	14(6.0)	2.44(0.76–8.03)	0.12
HIV status				
Negative	72(79.1)	233(89.3)	1	
Positive	19(20.9)	28(10.7)	2.19(1.15–4.19)	0.01
Cancer category				
Lymphomas	31(34.1)	121(46.4)	1	
Leukaemia’s	23(25.3)	71(27.2)	1.26(0.68–2.34)	0.45
Embryonal tumours	15(16.5)	34(13.0)	1.72(0.82–3.57)	0.13
Primary bone tumours	3(3.3)	11(4.2)	1.06(0.27–4.06)	0.92
Others	19(20.9)	24(9.2)	3.09(1.47–6.48)	< 0.001
Treatment duration				
0–12 months	72(79.1)	167(64.0)	1	
12–36 months	15(16.5)	88(33.7)	0.39(0.21–0.73)	0.002
> 36 months	4(4.4)	6(2.3)	1.54(0.42–5.6)	0.50
Relationship with caregiver				
Parent	46(50.4)	157(60.2)	1	
Relative	12(13.2)	30(11.5)	1.36(0.64–2.88)	0.43
Sibling	7(7.7)	19(7.3)	1.25(0.49–3.18)	0.62
Self (no relative/no caregiver)	23(25.3)	54(20.7)	1.45(0.80–2.62)	0.21
Other (friend/caretaker)	3(3.3)	1(0.4)	10.23(1.0–104.8)	0.01
Negative coping strategies				
Denial				
Yes	37(40.7)	104(39.8)	1.03(0.63–1.68)	0.89
No	54(59.3)	157(60.2)	1	
Self-blame				
Yes	20(22)	36(13.8)	1.76(0.95–3.24)	0.06
No	71(78)	225(86.2)	1	
Substance use				
Yes	3(3.3)	14(5.4)	0.60(0.16–2.14)	0.42
No	88(96.7)	249(94.6)	1	
Behavioural disengagement				
Yes	51(56)	122(46.7)	1.42(0.89–2.35)	0.12
No	40(44)	139(53.3)	1	
Positive coping strategies				
Self-distraction				
Yes	40(44)	210(80.5)	0.19(0.10–0.33)	< 0.001
No	51(56)	51(19.5)	1	
Active coping				
Yes	85(93.4)	244(93.5)	0.98(0.37–2.58)	0.97
No	6(6.6)	17(6.5)	1	
Use of emotional support				
Yes	72(79.1)	229(87.7)	0.52(0.28–0.99)	0.04
No	19(20.9)	32(12.3)	1	
Use of instrumental support				
Yes	73(80.2)	222(85.1)	0.71(0.38–1.32)	0.28
No	18(19.8)	39(14.9)	1	
Acceptance				
Yes	42(46.2)	148(56.7)	0.65(0.40–1.05)	0.08
No	49(53.8)	113(43.3)	1	
Positive reframing				
Yes	27(29.7)	176(67.4)	0.51(0.31–0.84)	0.07
No	44(70.3)	85(32.6)	1	
Use of religion				
Yes	63(69.2)	201(77)	0.67(0.39–1.14)	0.14
No	28(30.8)	60(23)	1	
Perception of social support				
Special person in patient’s life				
Yes	63(69.2)	229(87.7)	0.34(0.18–0.61)	0.002
No	28(30.8)	32(12.3)	1	
Emotional Support from family				
Yes	65(71.4)	228(87.4)	0.39(0.21–0.71)	0.001
No	26(28.6)	33(12.6)	1	
Friends in patient’s life				
Yes	64(70.3)	226(86.6)	0.36(0.20–0.65)	0.004
No	27(29.7)	35(13.4)	1	
Family help				
Yes	65(71.4)	227(87)	0.40(0.23–0.73)	0.001
No	26(28.6)	34(13)	1

**Table 4:** Bivariate logistic regression analyses of caregiver characteristics

Caregiver characteristic	Depression (*N* = 91) *n* (%)	No depression (*N* = 261) *n* (%)	OR (95% CI)	*P* Value
Marital status				
Single	4(4.4)	26(10)	1	
Married/cohabiting	66(72.5)	189(72.4)	2.26(0.75–6.78)	0.13
Divorced/separated	8(8.8)	25((9.6)	2.08(0.54–7.97)	0.27
Widowed	13(14.3)	21(8)	4.44(1.16–17.01)	0.01
Income				
< 75,000shs	32(35.2)	71(27.2)	1	
75,000-300,000	42(46.2)	131(50.2)	0.71(0.41–1.22)	0.21
300,000-500,000	11(12.1)	51(19.5)	0.47(0.21–1.04)	0.06
> 500,000	4(4.4)	4(1.5)	2.21(0.51–9.57)	0.27
Gender				
Male	26(28.6)	83(31.8)	0.85(0.50–1.45)	0.56
Female	65(71.4)	178(68.2)	1	
Employment				
Yes	70(76.9)	217(83.1)	0.65(0.37–1.21)	0.18
No	21(23.1)	44(16.9)	1	
Number of caregiver’s children				
0	3(3.3)	23(8.8)	1	
1–3	23(25.3)	73(28)	2.4(0.65–8.92)	0.17
4–6	36(39.6)	96(36.8)	2.87(0.80–10.32)	0.09
> 6	29(31.9)	69(26.4)	3.22(0.87–11.85)	0.06
Relationship with patient				
Parent	46(50.4)	157(60.2)	1	
Relative	12(13.2)	30(11.5)	1.36(0.64–2.88)	0.43
Sibling	7(7.7)	19(7.3)	1.25(0.49–3.18)	0.62
Self (no caregiver)	23(25.3)	54(20.7)	1.45(0.80–2.62)	0.21
Other (friend/caretaker)	3(3.3)	1(0.4)	10.23(1.0–104.8)	0.01

*OR* Odds ratio, *CI* Confidence interval, *N* Population size, *n* Sample size, *<* Less than, % Percent

**Table 5:** Multivariate logistic regression analyses

Characteristic	Adjusted OR (95% CI)	*P* value
Age		
7–9 years (children	1	
10–17 years (adolescents)	4.02(2.05–7.88)	< 0.001
Perception of social support		
A special person in the patient’s life		
No	1	
Yes	0.36(0.19–0.69)	0.002
Positive coping strategies		
Self-distraction		
No	1	
Yes	0.17(0.10–0.30)	< 0.001

*OR* Odds ratio, *CI* Confidence interval, *N* Population size, *n* Sample size, < Less than, % Percent

Significant bivariate logistic regression analyses include the following; adolescents were more likely to suffer from depression as compared to children, single orphans more likely to suffer from depression compared to non-orphans, HIV positive participants were more likely to suffer from depression compared to HIV the negative ones, those suffering from other type of cancers were more likely to suffer from depression compared to those with leukemia, those with a treatment duration of 12–36 months were less likely to suffer from depression as compared to those with a treatment duration of 0–12 months, those with a caregiver such as a friend or caretaker were more likely to suffer from depression compared to those taken care of by parents. Positive coping mechanism of self-distraction and emotional support plus a good perception of social support were protective against depression.

Significant bivariate logistic regression analyses include the following; children and adolescents whose parents had died were more likely to suffer from depression compared to those with a single parent, those taken care of by a friend or non-relative caretaker were also more likely to suffer from depression compared to those taken care of by a parent.

Both forward and backward stepwise regression models were run to determine factors independently associated with major depression, they include; having a supportive special friend, positive coping by use of self-distraction techniques and older age as shown in Table 5 below. Both stepwise regression models attained R-squared (R2) = 0.184 which indicates that the selected independent variables explained almost 18% of the variability of our dependent variable.

## Discussion

This study contributes to the research literature on the prevalence and factors associated with depression among children and adolescents with cancer in sub-Saharan Africa. The prevalence of major depressive disorder (MDD) was estimated at 26% among children and adolescents with cancer attending the Uganda Cancer Institute Out-patient clinic. This is quite substantial meaning that one in four children and adolescents at the UCI outpatient clinic (1/4) has major depression.

This finding is comparable to the findings of studies investigating depression in adults with cancer. For example, Atesci and colleagues conducted a study in Turkey in which the prevalence of major depression was estimated at 25% among 117 cancer patients using DSM-IV SCID. [4]. Furthermore, a review of studies reporting on the integration of mental health care into the Non-Communicable Disease Agenda reports a prevalence estimate of depression at 33% among adults with cancer [5].

Research studies on depression in children and adolescents with cancer are limited, and the few studies are concentrated in developed countries and report variable prevalence rates of depression. For example, Farhangi and colleagues conducted a study in 42 children and adolescents with Acute Lymphocytic Leukemia referred to pediatric haematology department of Dr. Sheikh hospital using CDI, they found a prevalence of 59.5% of depressive symptoms [13]. A possible explanation of this high rate could be that they assessed depression using a screening measure rather than a diagnostic interview.

Studies on the prevalence of MDD in individuals with cancer in our setting could not be found, therefore there is a need for such studies to be carried out. This is because findings from such studies will provide information that may be useful in directing policy and designing counselling intervention strategies that will help reduce depression and improve the overall prognosis of child and adolescent cancers. This is the first study to describe the prevalence of MDD in children and adolescents with cancer in our setting.

Adolescents (10–17 years) were four times more likely to suffer from MDD as compared to children. This is in line with study findings that indicate that the rate of major depressive episodes dramatically increases as children turn into adolescence [14, 15]. This could also be explained by the various hormonal changes happening during adolescence, the societal expectations during this age group and their ability to better conceptualize their emotions. This calls for intervention programs to be tailor-made to suit more of the adolescent population.

Notably, there wasn’t a significant association between MDD and the gender of the respondents, this is similar to findings of a study at Yale University that examined the emergence of gender difference in depression and found no gender differences in depression rates in prepubescent children [16].

Children and adolescents with a good perception of social support were less likely to suffer from MDD. These respondents felt that they had a special person in their lives and this protected them against MDD. This is in line with findings by a study conducted by Grav, Siv et al. [17] about association between social support and depression in the general population which showed that aspects of greater social support were related to less depressive symptomatology and social support was seen as one of the social determinants for overall health in the general population [18]. Another study by Barger Steven and colleagues about the social relationship correlates of major depressive disorder and depressive symptoms in Switzerland further demonstrated that perceived quality and frequency of social relationships are associated with clinical depression and depressive symptoms, as his finding indicated good perception of social support was protective against depressive disorders [17].

Use of self-distraction as a positive coping strategy protected the children and adolescents against MDD. This is in line with the findings of a meta-analysis of the effectiveness of strategies derived from the process model of emotion regulation which concluded that distraction was an effective way to regulate emotions [19].

This study had limitations. The first temporal relationship between cancer and MDD could not be established so was causal associations. This is because the outcome and exposure were measured at the same time. We would want to do a prospective study to establish a causal relationship. Second, the study sample consists of individuals at the clinic; therefore the findings can only be generalized to children and adolescents in a clinical setting. Third, there was a recall bias caused by differences in the accuracy or completeness of the recollections retrieved by study participants regarding events or experiences from the past. This is a methodological issue due to use of interviews or questionnaires.

Fourth, the measure for perceived social support was not comprehensive, never the less, it gave an indication of the level of support perceived by the children and adolescents. There is a need to conduct a full assessment of the measure of perceived social support in future studies.

Lastly, we would like to acknowledge that the size difference between the odds ratio of the protective and risk factors and the large confidence interval in some categories could be due to the small sample size of these categories.

Despite these limitations, this study to our knowledge provides the first prevalence estimates of depression symptoms among children and adolescents with cancer in Uganda. Our study has important implications for the management of children and adolescents with cancer; first training of the UCI staff in basic mental health care to enable them screen for mental health issues in the day to day care offered to the children and adolescents with cancer will ensure early diagnosis and intervention for mild to moderate depression thereby preventing the progression to severe depression. Prompt management of depression may lead to improved adherence to treatments and better cancer treatment outcomes. Second, knowledge of the factors associated with depression will guide the development of first-line psychological treatments for depression for both the affected individuals and their caregivers which can be integrated into the routine care of children with cancer.

## Conclusion

The prevalence of MDD in children and adolescents with cancer is substantial. Given that the majority had mild-moderate depression, first line psychological treatments for depression should be integrated into the routine care of children and adolescents with cancer.

## Abbreviations

CDI: Child Depression Inventory
DSM-IV: Diagnostic and Statistical Manual of Mental Disorders Fourth edition
HIV: Human immunodeficiency virus
MDD: Major depressive disorder
MINI-Kid: The Mini-International Neuropsychiatric Interview for Children and Adolescents
SCID: Structured Clinical Interview
UCI: Uganda Cancer Institute
